# Suppression effect of recombinant adenovirus vector containing hIL-24 on Hep-2 laryngeal carcinoma cells

**DOI:** 10.3892/ol.2014.1789

**Published:** 2014-01-10

**Authors:** XUEMEI CHEN, DI LIU, JUNFU WANG, QINGHONG SU, PENG ZHOU, JINSHENG LIU, MENG LUAN, XIAOQUN XU

**Affiliations:** 1 Department of Otolaryngology, The Second Affiliated Hospital of Shandong University, Jinan, Shandong 250033, P.R. China; 2 Institute of Basic Medicine, Shandong Academy of Medical Sciences, Jinan, Shandong 250062, P.R. China; 3 Medical Laboratory of the People’s Hospital of Tengzhou, Tengzhou, Shandong 277500, P.R. China

**Keywords:** human interleukin-24, adenovirus, Hep-2, apoptosis, human umbilical vein endothelial cell

## Abstract

The melanoma differentiation-associated gene-7 [MDA-7; renamed interleukin (IL)-24] was isolated from human melanoma cells induced to terminally differentiate by treatment with interferon and mezerein. MDA-7/IL-24 functions as a multimodality anticancer agent, possessing proapoptotic, antiangiogenic and immunostimulatory properties. All these attributes make MDA-7/IL-24 an ideal candidate for cancer gene therapy. In the present study, the human MDA-7/IL-24 gene was transfected into the human laryngeal cancer Hep-2 cell line and human umbilical vein endothelial cells (HUVECs) with a replication-incompetent adenovirus vector. Reverse transcription polymerase chain reaction and western blot analysis confirmed that the Ad-hIL-24 was expressed in the two cells. The expression of the antiapoptotic gene, Bcl-2, was significantly decreased and the IL-24 receptor was markedly expressed in Hep-2 cells following infection with Ad-hIL-24, but not in HUVECs. In addition, the expression of the proapoptotic gene, Bax, was induced and the expression of caspase-3 was increased in the Hep-2 cells and HUVECs. Methyl thiazolyl tetrazolium assay indicated that Ad-hIL-24 may induce growth suppression in Hep-2 cells but not in HUVECs. In conclusion, Ad-hIL-24 selectively inhibits proliferation and induces apoptosis in Hep-2 cells. No visible damage was found in HUVECs. Therefore, the results of the current study indicated that Ad-hIL-24 may have a potent suppressive effect on human laryngeal carcinoma cell lines, but is safe for healthy cells.

## Introduction

Laryngeal carcinoma is a common type of head and neck cancer with poor prognosis. The disease occurs mainly in adult males who abuse tobacco and alcohol and is characterized by squamous differentiation (1). Laryngeal carcinoma is usually identified in patients at late stage leading to reduced treatment efficacy and a high rate of recurrence. Despite the advances in the use of molecular markers for monitoring human cancer over the past decades, no reliable markers exist to screen laryngeal carcinoma and follow-up patients after treatment. Based on the structure, chromosomal location and biological/biochemical properties of the melanoma differentiation-associated gene-7 (MDA-7), it has now been classified as a novel member of the interleukin (IL)-10 gene family (2–4).

This tumor suppressor gene associated with differentiation, growth and apoptosis was initially identified from human melanoma cells (5,6). Mapped within the IL-10 family cytokine cluster to chromosome 1q32.2-q41, the gene encodes a protein consisting of 206 amino acids, secreted in mature form as a 35–40 kDa-phosphorylated glycoprotein (7,8).

MDA-7 is expressed by diverse cell types, including B cells, natural killer cells, dendritic cells, monocytes and melanocytes. Although its physiological role is poorly understood, the forced expression of MDA-7 in cancer cells results in irreversible growth inhibition, reversal of the malignant phenotype and terminal differentiation (9). Thus, the biological impact of MDA-7 on the behavior of laryngeal carcinoma cells was evaluated in the present study.

## Materials and methods

### Cells and main reagents

Hep-2 (ATCC, Manassas, VA, USA), the human laryngeal cancer cell line and 293A, a subclone of the 293 cell line, were preserved at the Key Laboratory for Modern Medicine and Technology of Shandong Province (address?) and maintained in RPMI 1640 supplemented with 10% heat-inactivated fetal calf serum. Human umbilical vein endothelial cells (HUVECs) were obtained from the umbilical vein of healthy adults. The Ethics Committee of Shandong University School of Medicine approved the study and all patients provided written informed consent. Recombinant Ad-hIL-24 was constructed and the total RNA extract kit was produced by our laboratory. M-MLV reverse transcriptase and Taq DNA polymerase were purchased from Promega Corporation (Madison, WI, USA). Methyl thiazolyl tetrazolium (MTT) was purchased from Sigma-Aldrich (St. Louis, MO, USA) and RPMI-1640 was purchased from Gibco-BRL (Carlsbad, CA, USA). Serum from newborn calf was obtained from Hangzhou Sijiqing Biological Engineering Materials Co., Ltd. (Hangzhou, China). Human IL-24 monoclonal antibody was purchased from Abcam (Cambridge, UK), human Bcl-2 monoclonal antibody was purchased from Trevigen, Inc. (Gaithersburg, MD, USA), human Bax polyclonal antibody was purchased from Beijing Biosynthesis Biotechnology Co., Ltd. (Beijing, China), human caspase-3 monoclonal antibody was purchased from Bioworld Technology, Inc. (St. Louis Park, MN, USA) and actin polyclonal antibody was purchased from Santa Cruz Biotechnology, Inc. (Santa Cruz, CA, USA). Horseradish peroxidase-labeled goat anti-rabbit and anti-mouse IgG were purchased from Beijing Zhongshan Golden Bridge Biotechnology Co., Ltd. (Beijing, China).

### Recombinant adenovirus amplification and titer determination

The 70% adherent 293A cells were infected with Ad-hIL-24 or empty adenovirus (Ad-GFP) and collected following 48 h. The cell suspension was frozen and thawed three times at −80 and 37°C, respectively. The supernatant was then removed, infections were repeated and the cells were amplified. The virus solution was stored at −80°C.

For virus titer determination, 1×10^5^ 293A cells/ml were seeded in 96-well plates (100 μl/well) and cultured under 5% CO_2_ at 37°C for 24 h. The virus stock solution was then diluted from 1:10 to 1:10^10^ with 2% fetal bovine serum cell culture fluid. Then, 100 μl of 1:10^3^ to 1:10^10^ dilutions of the virus were added in the 96-well plates. In total, three wells were infected for each dilution of virus and the negative control was set. The 96-well plates were cultured at 37°C in a 5% CO_2_ incubator and the cytopathic effect was observed every day. After 96 h (4 days), >50% and <50% lesion well virus dilution were recorded in order to calculate the 50% tissue culture infective dose (TCID_50_) and subsequently calculate the PFU using the formula: Virus titer (pfu/ml) = 0.7 × TCID_50_.

### Identification of exogenous hIL-24 mRNA and protein in Hep-2 cells and HUVECs

Hep-2 cells and HUVECs were seeded in 6-well plates (2×10^5^/well) and then treated with phosphate-buffered saline (PBS) without calcium and magnesium ions or 100 multiplicity of infection (MOI) of Ad-GFP or 100 MOI of Ad-hIL-24 following 24 h. The cells were collected following culture at 37°C in a 5% CO_2_ incubator for 48 h.

The sequences of the IL-24 and β-actin primers are listed in Table I. β-actin controls were designed to be 18–24 nucleotides in length and to have 100% homology with particular regions of the gene. The gene sequences were obtained using the Oligo Primer analysis software, version 5.0 (NBA; Software and Research Services for Tomorrow’s Discoveries; National Biosciences, Inc., Plymouth, MN, USA) and polymerase chain reaction (PCR) oligomers were synthesized by a DNA/RNA synthesizer (Applied Biosystems, Inc., Foster City, CA, USA) at the BioSune Biotechnology (Shanghai) Co., Ltd. (Shanghai, China). The reverse transcription (RT)-PCR method was used as previously described (10). Briefly, RNA was extracted from tissues using the acid guanidinium phenol-chloroform method. The quality of the RNA yield was assessed by electrophoresis (EC250–90, E-C Apparatus Corporation, Milford, MA, USA) on a 1.5% agarose gel in 0.5 M Tris/borate/EDTA buffer, demonstrating the typical 28S and 18S bands of the total RNA in all RNA yielded from the cells. The amount of each RNA sample was measured by optical density reading and only RNA samples showing a A260-A280 ratio between 1.8 and 2.0 were used to obtain complementary DNA (cDNA). RT-PCR was performed using RNA PCR kit (Promega Corporation). Cell RNA (1 μg) was reverse transcribed into cDNA in a reaction mixture containing 1X buffer, 1 mM dNTP, 2.5 μM oligo (dT) primer, 1 unit RNAse inhibitor and 2.5 units reverse transcriptase. Following incubation at 37°C for 60 min, the reaction was terminated by heating at 95°C for 5 min. PCR was performed using the forward and reverse primers described in Table I. The PCR reaction buffer (25 μl), consisting of 2 mM MgCl_2_, 0.5 μM of each primer and 2 units AmpliTaq DNA polymerase (2 μl of each reverse-transcriptase solution) was added to an amplification tube. PCR was run for 33 cycles and each cycle consisted of 95°C for 1 min, 55°C for 1 min and 72°C for 1 min, followed by a final extension for 7 min. In total, 12 μl aliquots of the amplified product was fractionated on a 1.5% agarose gel and visualized by ethidium bromide staining. The band intensity of ethidium bromide fluorescence was measured using NIH/1D image analysis software version 1.61 (National Institutes of Health, Bethesda, MD, USA). The relative intensity of each band was determined by the ratio to β-actin. To exclude the possibility of carry-over contamination, reactions containing all the RT-PCR reagents, including cytokine PCR primers without sample RNA, were used as negative controls. No contamination was detected.

SDS-PAGE and immunoblotting was performed as previously described in the legend to each figure using standard techniques. In brief, the prepared cells were lysed at 4°C for 30 min in lysis buffer [20 mM tris(hydroxymethyl)aminomethane-HCl (pH 7.5), 140 mM NaCl, 1 mM ethylenediaminetetraacetic acid, 50 U/ml aprotinin, 1 mM phenylmethylsulfonyl fluoride and 1 mM sodium orthovanadate] containing 1% Nonidet P-40 detergent (11) and the protein samples were boiled for 10 min. The boiled samples were loaded onto a 14% SDS-PAGE gel and electrophoresis was run for 2 h. Proteins were electrophoretically transferred onto 0.22 μm nitrocellulose membrane and immunoblotted with IL-24 monoclonal and β-actin antibodies against different proteins. The immunoblots were visualized using a LAS4000 Chemiluminescence Imager (Fijifilm, Tokyo, Japan) with associated software. For presentation, immunoblots were opened in PhotoShop CS2 (Adobe Systems, Mountain View, CA, USA); the color was removed and figures were generated in PowerPoint (Microsoft Corporation, Redmond, WA, USA).

### Cytotoxicity of Ad-hIL-24

Hep-2 cells and HUVECs were seeded in culture plates, 24 h following the addition of PBS without calcium and magnesium ions or infection with 100 MOI of Ad-GFP or 100 MOI of Ad-hIL-24. The cells were cultured at 37°C in a 5% CO_2_ for 48 h. Morphological changes were observed under an inverted fluorescence microscope (IX70, Olympus, Tokyo, Japan).

### Ad-hIL-24 effect on cell growth by MTT assay

Hep-2 cells and HUVECs were inoculated in 96-well plates, separately, at 100 μl/well (5×10^4^/ml). The cells were divided into three groups following cell adherence and the assay was repeated three times for each group. The cells were added to PBS or infected with 100 MOI of Ad-GFP or 100 MOI of Ad-hIL-24 (100 μl/well) and observed for four days. A total of 10 μl MTT (5 mg/ml) was added to each well of the three groups every 24 h and incubated at 37°C for 4 h. Then, 100 μl SDS-HCl (10%) stopping solution was added to each well to fully dissolve the formazan particles. The groups were measured with a microplate reader at 570 nm wavelength absorbance (A) and a growth curve of the time effect was drawn with the A value as the vertical axis and incubation time as the abscissa.

### IL-24 effect on Bcl-2, Bax, caspase-3 and IL-24 receptor mRNA expression in Hep-2 cells and HUVECs by RT-PCR

IL-24 receptor includes IL-20R1, IL-20R2 and IL-22R. IL-20R1 and IL-22R were selected as the IL-24 receptors to detect expression in Hep-2 cells and HUVECs. The sequences of Bcl-2, Bax, caspase-3, IL-20R1 and IL-22R primers are listed in Table I. Cell preparation, RNA extraction, reverse transcription and PCR were performed as described above.

### IL-24 effect on Bcl-2, Bax and caspase-3 protein expression in Hep-2 cells and HUVECs by western blot analysis

Hep-2 cells and HUVECs were seeded separately in culture plates. Following 24 h, the cells were added to PBS or infected with 100 MOI of Ad-GFP or 100 MOI of Ad-hIL-24. The cells were then incubated at 37°C and 5% CO_2_ for 48 h, digested with trypsin and collected. SDS-PAGE and immunoblotting were performed as previously described. Proteins were electrophoretically transferred onto 0.22 μm nitrocellulose membranes and immunoblotted with various primary antibodies (Bcl-2, Bax, caspase-3 and β-actin) against different proteins. Immunoblots were visualized using a LAS4000 Chemiluminescence Imager (Fijifilm) with associated software.

### Statistical analysis

Comparison of the effects of various treatments was performed using one-way analysis of variance (ANOVA) using the statistical software SPSS 11.5 (SPSS, Inc., Chicago, IL, USA). P<0.05 was considered to indicate a statistically significant difference.

## Results

### Amplification and titer determination of the recombinant adenovirus

Following infection of 293A cells with Ad-GFP or Ad-hIL-24 for 24 h, green fluorescence was observed in the cells under an inverted fluorescence microscope. Determination of the amplified adenovirus by the TCID_50_ method demonstrated that the titer of recombinant adenovirus was 7×10^8^ pfu/ml following multiple rounds of amplification.

### Identification of exogenous hIL-24 mRNA and protein in Hep-2 cells and HUVECs

**Figure 1:**
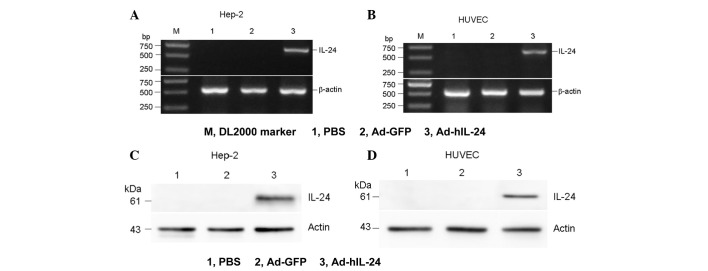
Exogenous hIL-24 messenger RNA and protein expression in Hep-2 cells and HUVECs. Total RNA and protein were obtained from Hep-2 cells and HUVECs infected with Ad-hIL-24 or Ad-GFP, serving as a blank adenovirus control or untreated cells, respectively. (A and B) First-strand complementary DNA was synthesized from RNA using reverse transcription. Polymerase chain reaction was conducted using primer sets specific for IL-24 and the housekeeping gene, β-actin, was used as an internal control. (C and D) Western blot analysis detected IL-24 protein expression in Hep-2 cells and HUVECs. HUVECs, human umbilical vein endothelial cells; IL, interleukin; PBS, phosphate-buffered saline.

The Ad-hIL-24 group was found to exhibit a specific DNA band at the 500–750-bp position and a protein band at the 51-kDa position, while the PBS and Ad-GFP groups did not show any bands. This finding indicated that the adenovirus-mediated hIL-24 gene and protein was successfully transcripted and translated in the Hep-2 and HUVECs, respectively (Fig. 1).

### Cytotoxicity of Ad-hIL-24

**Figure 2:**
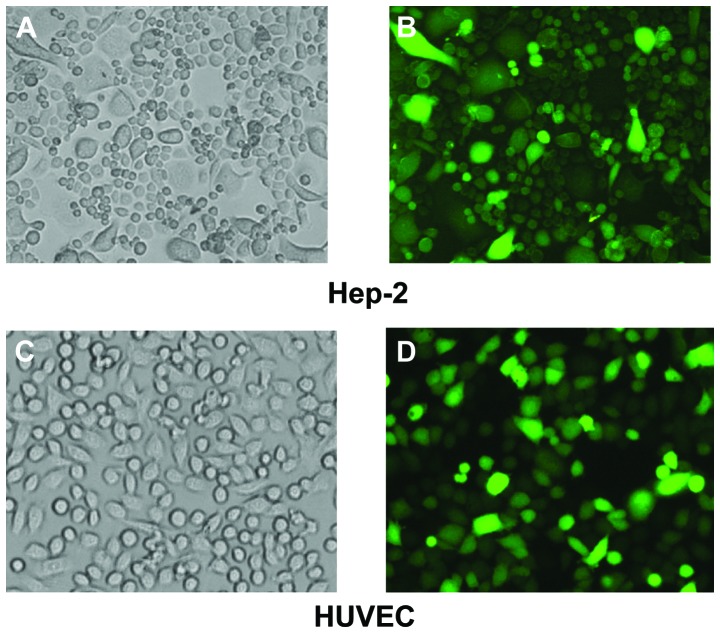
Morphological changes in Hep-2 cells and HUVECs infected with Ad-hIL-24. Hep-2 cells infected with Ad-hIL-24 at 48 h under (A) ordinary optical and (B) fluorescence microscopy. HUVECs infected by Ad-hIL-24 at 48 h under (C) ordinary optical and (D) fluorescence microscopy (magnification, ×200). HUVECs, human umbilical vein endothelial cells.

Under the microscope the living Hep-2 cells were observed to adhere to the culture plate and were fusiform in shape. Following 48 h the Ad-hIL-24-infected cells underwent apoptosis and the cell shape became rounder and the cells detached from the plate. Subsequently, the cell membranes shrank and the cells ruptured. Hep-2 cells treated with PBS and Ad-GFP and HUVECs treated with Ad-hIL-24, PBS and Ad-GFP did not show these changes (Fig. 2).

### Ad-hIL-24 effect on cell growth by MTT assay

**Figure 3:**
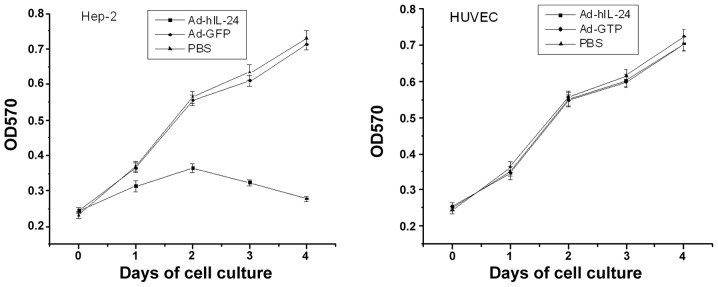
Time effect of Ad-hIL-24 on Hep-2 cells and HUVECs. Hep-2 cells and HUVECs were treated with Ad-hIL-24 at a multiplicity of infection of 100 or with Ad-GFP or PBS, serving as controls for four days. The survival of cells was evaluated on days 0, 1, 2, 3 and 4 following infection by methyl thiazolyl tetrazolium assay. The growth of Hep-2 tumor cells treated with Ad-hIL-24 was significantly inhibited following infection (P<0.05, vs. Ad-GFP and PBS groups at days 2, 3 and 4), but was not significantly inhibited in the Ad-GFP group (P>0.05, vs. PBS group, via ANOVA). In addition, Ad-hIL-24 had no effect on HUVECs (P>0.05, vs. Ad-GFP and PBS groups, via ANOVA). Experiments were repeated three times per condition. HUVECs, human umbilical vein endothelial cells; PBS, phosphate-buffered saline; ANOVA, one-way analysis of variance; OD, optical density.

Hep-2 cell proliferation was significantly inhibited following infection with Ad-hIL-24 and indicated a time-dependent trend. Cell proliferation was significantly different between the Ad-hIL-24-treated, PBS control or Adv-treated groups by ANOVA (P<0.01). No statistically significant difference was identified between the PBS control and Adv-treated groups (P>0.05; Fig. 3). These results showed that Ad-MDA-7/IL-24 inhibited the proliferation of laryngeal cancer cells. In addition, no change was identified between the Ad-hIL-24-treated, PBS control or Adv-treated groups (P>0.05) in HUVECs.

### RT-PCR detection of the mRNA of related apoptosis molecules

**Figure 4:**
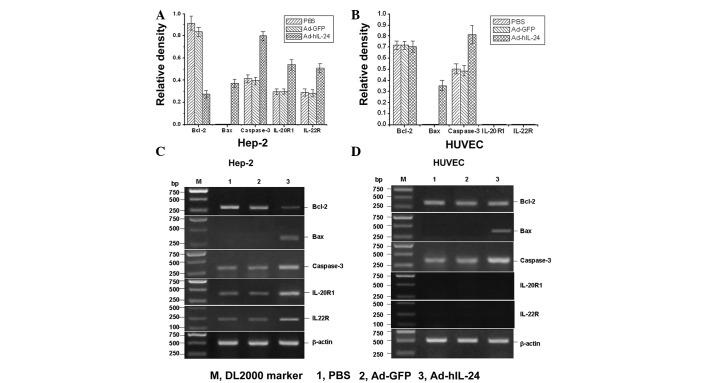
Reverse transcription polymerase chain reaction analysis of the mRNA expression of apoptosis-related genes and the IL-24 receptor. Average mRNA expression of Bcl-2, Bax, caspase-3, Il-20R1 and IL-22R in (A) Hep-2 cells and (B) HUVECs. All experiments were repeated twice and each experiment was performed in triplicate for each sample. (C) Gel electrophoresis of the mRNA expression of Bcl-2, Bax, caspase-3, Il-20R1 and IL-22R in Hep-2 cells. IL-24 induced the proapoptotic gene Bax expression and increased caspase-3, IL-20R1 and IL-22R mRNA expression and antiapoptotic gene Bcl-2 expression was significantly reduced in Hep-2 cells. (D) Gel electrophoresis of the mRNA expression of Bcl-2, Bax, caspase-3, Il-20R1 and IL-22R in HUVECs. The Bax and caspase-3 expression levels were similar to that of Hep-2 cells, but Bcl-2 expression did not change and no expression of IL-20R1 and IL-22R was identified. mRNA, messenger RNA; IL, interleukin; HUVECs, human umbilical vein endothelial cells; PBS, phosphate-buffered saline.

The mRNA expression of apoptosis-related molecules, Bcl-2, Bax and caspase-3, was detected by RT-PCR assay. The results showed that IL-24 induced proapoptotic gene Bax expression and increased caspase-3 mRNA expression. Antiapoptotic gene Bcl-2 expression was significantly decreased while the IL-24 receptor was markedly expressed in Hep-2 cells. In HUVECs, the Bax and caspase-3 expression was similar to that of Hep-2 cells, but Bcl-2 expression did not change and no expression of the IL-24 receptor was identified (Fig. 4). This result showed that IL-24 inhibits antiapoptotic genes and increases the expression of apoptotic genes to promote tumor cell apoptosis. In addition, IL-24 also enhanced the expression of the IL-24 receptor, thus, promoting apoptosis in Hep-2 cells.

### Western blot analysis detection of the protein of related apoptosis molecules

**Figure 5:**
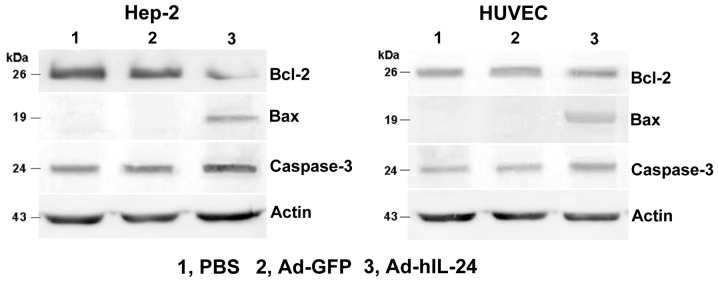
Western blot analysis of the apoptosis-related protein expression map. Hep-2 cells and HUVECs were cultured with Ad-hIL-24, Ad-GFP or PBS for 48 h and their cell lysate was subjected to western blot analysis for the detection of Bcl-2, Bax, caspase-3 and β-actin (used as an internal control) expression. Hep-2 cells treated with Ad-hIL-24 expressed significantly reduced levels of Bcl-2 than those in the Ad-GFP and PBS groups, but no change was identified in HUVECs. Hep-2 cells and HUVECs treated with Ad-hIL-24 expressed significantly higher levels of caspase-3 than those in the Ad-GFP and PBS groups. In addition, Ad-hIL-24 induced the activation of Bax in Hep-2 cells and HUVECs. Data shown are representative of three independent experiments. HUVECs, human umbilical vein endothelial cells; PBS, phosphate-buffered saline.

The protein expression of apoptosis-related molecules, Bcl-2, Bax and caspase-3, was analyzed by western blot analysis. The results revealed that IL-24 induced proapoptotic gene Bax protein expression and increases caspase-3 protein expression. Antiapoptotic gene Bcl-2 protein expression was significantly reduced in Hep-2 cells. In HUVECs, the Bax and caspase-3 protein expression was similar to that of Hep-2 cells, but Bcl-2 protein expression did not change (Fig. 5). This showed that IL-24 inhibited the expression of the antiapoptotic protein and increased the expression of the apoptotic protein to promote tumor cell apoptosis.

## Discussion

MDA-7/IL-24 was identified by subtraction hybridization strategy in the mid-1990s (5). The MDA-7 gene was isolated from human melanoma cells induced to terminally differentiate by treatment with interferon and mezerein. The protein expression of MDA-7/IL-24 is decreased during melanoma progression, with almost imperceptible levels in metastatic disease (5,6,12,13). MDA-7/IL-24 has been mapped within the IL-10 family cytokine cluster to 1q32.2-q41 and the gene encodes a protein consisting of 206 amino acids, secreted in mature form as a 35–40 kDa-phosphorylated glycoprotein (7,8).

One of the essential requirements of utilizing a therapeutic gene in gene therapy is that its expression must not induce any deleterious effects in normal cells. Therefore, MDA-7/IL-24 fits the requirements of a therapeutic gene. Previous studies analyzing MDA-7/IL-24 have clearly shown the absence of deleterious effects on normal human cells, including normal melanocytes, endothelial cells, astrocytes, mammary and prostate epithelial cells and skin fibroblasts (9,14–18).

MDA-7/IL-24 is a potent therapeutic cancer gene due to its broad-spectrum cancer-specific apoptosis-inducing properties as well as its multipronged indirect antitumor activities (19). Although its physiological role is poorly understood, forced expression of MDA-7 in cancer cells results in irreversible growth inhibition, reversal of the malignant phenotype and terminal differentiation (9). Previous *in vitro* and *in vivo* studies have demonstrated these attributes to be tumor-selective and applicable to numerous solid malignancies. The ectopic expression of MDA-7 (by transfection or adenovirus transduction) exerts potent growth-suppressive and apoptosis-inducing effects, not only in human melanoma cells, but also in a wide spectrum of human cancer cells, including malignant glioma, osteosarcoma, mesothelioma and carcinomas of the breast, cervix, colon, lung, ovary and prostate (2–4,14,16,20). Notably, similar effects are not apparent following transduction into their non-malignant counterparts (18). Specific antitumor activity has also been established in a range of human tumor xenograft models and in several early phase clinical trials involving patients with advanced solid cancers (2,20–22). MDA-7 is emerging as a differentiation-, growth- and apoptosis-associated gene with potential utility for the gene-based therapy of several types of human cancer (7).

The apoptotic pathways by which MDA-7/IL-24 kills tumor cells remain to be fully understood; however, current evidence suggests an inherently high degree of complexity and an involvement of proteins important for the onset of growth inhibition and apoptosis, including Bcl-XL, Bcl-2 and Bax (3,4,14,17,23–25). MDA-7 has also been shown to influence endothelial cells, exerting a potentially antiangiogenic effect within the tumor vasculature (26). Ad-MDA-7 has been found to mediate p53-independent inhibition of tumor growth, cell cycle arrest and apoptosis, associated with the downregulation of Bcl-2 and Akt. In previous *in vivo* studies, growth inhibition has been demonstrated in multiple xenograft models. Furthermore, Ad-MDA-7 has been demonstrated to have an additive or synergistic effect in cellular and animal studies when combined with chemotherapy, biological therapies and radiotherapy. These effects have been associated with a decreased Bcl-2 expression and Bax upregulation (27).

Laryngeal carcinoma, one of the most common tumors of the head and neck, occurs mainly in adult males who abuse tobacco and alcohol and is characterized by squamous differentiation. Although early-stage glottic cancer has a favorable prognosis, with five-year survival rates of >70% (1), numerous types of supraglottic and subglottic cancer are not diagnosed until severe signs develop, by which time the five-year survival rate has decreased to <50%. Locoregional recurrence, cervical lymph node metastases and distant metastases are the factors significantly affecting prognosis in laryngeal squamous carcinoma patients (28). The recognition and identification of tumor markers associated with recurrence and/or metastasis are key elements in predicting the biological behavior of the tumor and deciding on the most appropriate therapeutic strategy.

MDA-7 induces cell cycle arrest at the G2/M phase, induces apoptosis in cancer cells, inhibits new blood vessel formation essential for tumor growth and stimulates the immune system. In addition, MDA-7 is a secreted protein, which allows it to exhibit bystander effects resulting in amplified tumor cell killing.

In the present study, the human MDA-7/IL-24 gene was transfected into the human laryngeal cancer Hep-2 cell line and HUVECs with a replication-incompetent adenovirus vector. The expression of Bcl-2 was significantly decreased while the IL-24 receptor was markedly expressed in Hep-2 cells following infection with Ad-hIL-24, but not in HUVECs. In addition, the expression of Bax and caspase-3 was increased in Hep-2 cells and HUVECs. This finding showed that IL-24 inhibits antiapoptotic genes and increases the expression of apoptotic genes to promote tumor cell apoptosis. Furthermore, IL-24 also enhances the expression of the IL-24 receptor, thus, stimulating apoptosis in Hep-2 cells. Bcl-2 expression did not change and no expression of the IL-24 receptor was identified in the HUVECs. In addition to the IL-24 receptor, other methods may exist that enhance the increased expression of Bax and caspase-3. The MTT assay of the present study indicated that Ad-hIL-24 induces growth suppression in Hep-2 cells but not in HUVECs. Therefore, the results have shown that Ad-hIL-24 selectively inhibits proliferation and induces apoptosis of Hep-2 cells. No visible damage was identified in the normal cells under the microscope. Therefore, the present study, evaluating MDA-7vIL-24 in the context of laryngeal carcinoma, may prove to be extremely valuable for developing an effective gene therapy strategy for laryngeal carcinoma.

## Figures and Tables

**Table I:** Oligonucleotide-specific primers used to demonstrate associated gene messenger RNA expression in Hep-2 cells and HUVECs.

Target gene	Oligonucleotide sequence	Length (bp)
β-actin		
F	5′-gtggggcgccccaggcacca-3′	539
R	5′-ctccttaatgtcacgcacgattt-3′	
IL-24		
F	5′-tactcgagagatgaattttcaacagaggct-3′	621
R	5′-gcgtctagatatcagagcttgtagaat-3′	
Bcl-2		
F	5′-cgacgacttctcccgccgctaccgc-3′	319
R	5′-ccgcatgctggggccgtacagttcc-3′	
Bax		
F	5′-tccaccaagaagctgagcgag-3′	355
R	5′-gtccagcccatgatggttct-3′	
Caspase-3		
F	5′-cccatttctccatacgcact-3′	358
R	5′-tgacagccagtgagacttgg-3′	
IL-20R1		
F	5′-tcaaacagaacgtggtcccagtg-3′	386
R	5′-tccgagatattgagggtgataaag-3′	
IL-22R		
F	5′-ccccactgggacactttcta-3′	243
R	5′-tggccctttaggtactgtgg-3′	

HUVECs, human umbilical vein endothelial cells; F, forward; R, reverse; IL, interleukin.
